# The effect of colchicine on micronutrients in children with newly diagnosed familial Mediterranean fever

**DOI:** 10.1016/j.jped.2026.101554

**Published:** 2026-05-23

**Authors:** Özge Kaba, Ayşe Tanatar, Rahime Koç, Şerife Gül Karadağ, Mustafa Çakan, Nuray Aktay Ayaz

**Affiliations:** a Başakşehir Çam and Sakura City Hospital, Department of Pediatrics, Division of Pediatric Infectious Diseases, Istanbul, Turkey; b Marmara University Pendik Training and Research Hospital, Department of Pediatrics, Division of Rheumatology, Istanbul, Turkey; c Başakşehir Çam and Sakura City of Hospital, Department of Pediatrics, Division of Rheumatology, Istanbul, Turkey; d Bakırkoy Dr. Sadi Konuk Training and Research Hospital, Department of Pediatrics, Division of Rheumatology, Istanbul, Turkey; e Zeynep Kamil Training and Research Hospital, Department of Pediatrics, Division of Rheumatology, Istanbul, Turkey; f Istanbul University Istanbul Medical Faculty, Department of Pediatrics, Division of Rheumatology, Istanbul, Turkey

**Keywords:** Anemia, Colchicine, Familial Mediterranean Fever, Vitamin B_12_ deficiency

## Abstract

**Objectives:**

Colchicine is an essential component of familial Mediterranean fever (FMF) treatment. It is suggested that it may affect vitamin B_12_ levels by affecting intestinal absorption. This research focused on examining the impact of colchicine therapy on anemia parameters, as well as levels of vitamin B_12_ and folate in patients who have recently been diagnosed with FMF.

**Method:**

This prospective cohort investigation assessed children diagnosed with FMF between October 2016 and February 2017, evaluating anemia parameters as well as vitamin B_12_ and folate levels at baseline and at the third and sixth months of colchicine therapy. The demographic data were recorded from patient files.

**Results:**

Forty-six children were involved. M694V was the most common mutation. The most common clinical findings were fever (87%), peritonitis (76.1%), and pleuritis (23.9%). The mean hemoglobin (*p* 0.027), iron (*p* < 0.001) levels were significantly increased, while the mean ferritin (*p* 0.002), vitamin B_12_ (*p* < 0.001) and folate (*p* 0.001) levels were decreased at the 6^th^ month of colchicine treatment.

**Conclusion:**

Although it has been determined that the suppressive effect of colchicine on inflammation improves anemia by increasing hemoglobin levels, it should also be kept in mind that it may cause a decrease in the levels of other variables that can cause anemia, such as vitamin B_12_ and folate.

## Introduction

Familial Mediterranean Fever (FMF) is a typical autoinflammatory disease that causes episodes of increased inflammation, which clinically present as fever, polyserositis, and arthritis [1]. However, subclinical inflammation may persist despite attack-free periods. Due to the relapsing and remitting nature of the disease, chronic anemia may also be observed in children with FMF.

In addition to anemia of chronic disease, functional iron deficiency anemia can also occur due to altered iron metabolism caused by inflammatory factors such as hepcidin [2]. Levels of selected micronutrients, including folate and vitamin B_12_, which are important for growth and development, may also be affected by chronic inflammation, leading to decreased nutrient absorption and increased metabolic demands, thus contributing to anemia.

Colchicine is the standard treatment modality in FMF [3]. Research suggests that long-term daily colchicine use significantly diminishes attack frequency, severity, and duration, thereby preventing the development of amyloidosis [4]. However, colchicine may cause various adverse effects, especially in rapidly proliferating tissues such as the gastrointestinal tract and bone marrow. Specifically, colchicine has been associated with leukopenia, thrombocytopenia, and aplastic anemia due to its suppressive effect on bone marrow function [5]. These side effects are rarely observed and can often be managed by lowering the dosage [6]. Folate and vitamin B_12_, another key cofactor in cell replication and erythropoiesis, may also be depleted in chronic inflammatory states. Moreover, diarrhea, a common undesirable effect of colchicine, can also cause malabsorption and micronutrient deficiencies such as iron, vitamin B_12_ and folate. Besides, studies in both humans and animals indicate that colchicine may interfere with vitamin B_12_ uptake as a result of disruption of the ileal epithelium [7].

This study aims to evaluate vitamin B_12_ and folate levels, along with markers of iron homeostasis, in children newly diagnosed with FMF, and to investigate whether colchicine therapy is associated with deficiencies in these essential micronutrients.

## Materials and methods

This prospective study was conducted within the pediatric rheumatology unit of a tertiary referral hospital between October 2016 and February 2017. Children with newly diagnosed FMF who fulfilled the Tel-hashomer criteria were included in the study [8]. Demographic data, clinical features, and treatments were documented from medical charts. Patients included in the study were confirmed to be fully compliant with colchicine therapy; those with poor adherence were excluded. Patients were evaluated for serum iron, total iron binding capacity, ferritin, vitamin B_12_, and folate values before the initiation of colchicine treatment, then at the third and sixth months of colchicine treatment. The colchicine dose was adjusted according to the age of the patient (0.5 mg/day for 0–5 years, 1 mg/day for 5–10 years, and 1.5 mg/day over 10 years). Erythrocyte sedimentation rate (ESR), white blood cell count (WBC), C-reactive protein (CRP), hemoglobin (Hb), hematocrit (Hct), and serum amyloid A (SAA) values were recorded in every visit. Patients with coexistent disease or initial abnormal serum iron, total iron binding capacity, vitamin B_12_, or folate values were excluded from the study.

Among the cases constituting the study group, 9 mL blood sample was taken into a 1 mL 0.5 M Ethylene permanent tetra acetic acid (EDTA) (Sigma, USA) tube; DNA was obtained from the samples prepared by mixing RBC (Red Blood Cell) [155 mM Ammonium Chloride (Appli Chem, Germany), Sodium Bicarbonate (Merck, Germany), EDTA (Appli Chem, Germany)] and Proteinase K enzyme (MBIFermentas, Lithuania). For the 2^nd^, 3^rd^, 5^th^, and 10^th^ exon regions of the *MEFV* gene, an automated DNA sequence analysis device (Hitachi 3130XL, United States) was used, which operates based on Sanger's enzymatic sequencing method.

Complete blood count tests were performed by the SYSMEX (Hamburg, Germany) brand device; vitamin B_12_, folate, and ferritin parameters by chemoluminescence method; iron and total iron binding capacity were studied with COBAS 6000 (Roche, USA) and COBAS 8000 (Roche, USA) brand devices with spectrophotometric methods. In the studied serum samples, 200 pg/mL and below for vitamin B_12_ and 2 ng/mL and below for folate were considered deficiencies [9].

Approval for the study was granted by the local Ethics Committee (decision no 2016/8; September 27, 2016), and all procedures adhered to the principles set forth in the Declaration of Helsinki. All participants were enrolled after written informed consent had been provided by their legal guardians.

The SPSS (Statistical Package for Social Sciences) Windows 19.0 package program was used for statistical evaluations. Continuous variables were expressed as mean and standard deviation, while categorical variables were presented as counts and percentages. Categorically, the differences between the groups in terms of the frequencies of the data were examined by the chi-square test. The differences in mean scores between the groups were analyzed using the Mann–Whitney U test. All means with normal distribution are repeatedly measured with one-way analysis of variance and Bonferroni analysis; those without normal distribution were compared by Friedman analysis and the Wilcoxon signed rank test. For all statistical data, *p* < 0.05 was considered significant.

## Results

The study included 46 children with FMF [28 male (60.9 %), 18 female (39.1 %)]. The mean age of the patients was 100.6 ± 49.7 months, with symptom onset and diagnosis occurring at 65.3 ± 45.5 months and 97.1 ± 50.2 months, respectively. More than half of the patients (*n* = 25, 54.3 %) had a documented family history of FMF. The M694V mutation was the predominant *MEFV* variant, identified in 19 cases (40.8 %). Assessment of patients’ symptoms during attack periods revealed that fever occurred in 40 individuals (86.9 %), peritonitis in 35 (76 %), pleuritis in 11 (23.9 %), and arthritis in 6 (13 %).

Comparing pre-treatment and third-month data, a non-significant increase in hemoglobin (*p* 0.288) and a statistically significant increase in iron (*p* 0.007) were observed. Statistically significant decreases were observed in platelet count (*p* < 0.001), ferritin (*p* 0.001), vitamin B_12_ (*p* 0.013), folate (*p* 0.008), and SAA (*p* < 0.001). A comparison of the third and sixth months of treatment revealed a non-significant increase in hemoglobin (*p* 0.005), iron (*p* 0.307), and folate (*p* 0.893), and a non-significant decrease in platelet count (*p* 0.096), ferritin (*p* 0.170), and SAA (*p* 0.184). In the second 3-month period, a statistically significant decrease was observed only in serum vitamin B_12_ levels (*p* 0.004).

When pre-treatment values were compared with those at the end of the sixth month, a statistically significant increase was observed in hemoglobin (*p* 0.027) and iron (*p* < 0.001) levels, while a statistically significant decrease was observed in platelet count (*p* < 0.001), ferritin (*p* 0.002), vitamin B_12_ (*p* < 0.001), folate (*p* 0.001) and SAA (*p* < 0.001) levels. Table 1 presents the pre-treatment, third-month, and sixth-month treatment data for the aforementioned parameters.

**Table 1:** The comparison of laboratory parameters before starting treatment, at the 3rd and 6th months of colchicine treatment.

	**Pre-treatment**	**3^rd^ month of colchicine treatment**	**6^th^ month of colchicine treatment**	p*
	Mean ± SD (median)	Mean ± SD (median)	Mean ± SD (median)	
**Hemoglobin (g/dL)**	12.1 ± 1.1 (12.0)	12.2 ± 1.0 (12.0)	12.3 ± 1.0 (12.3)	0.027^a^
**Hematocrit (%)**	38.0 ± 3.1 (38.0)	38.6 ± 3.4 (38.3)	38.8 ± 2.8 (38.2)	0.135^b^
**Platelet**	350,932 ± 110,096 (334,600)	310,671 ± 85,475 (309,850)	303,202 ± 81,570 (296,700)	<0.001^b^
**SAA (mg/dL)**	192.1 ± 295.1 (38.9)	20.2 ± 46.5 (3.8)	9.6 ± 25.8 (3.3)	<0.001^b^
**Iron (µg/dL)**	53.4 ± 29.5 (50.8)	66.7 ± 35.6 (56.5)	67.4 ± 31.1 (64.0)	<0.001^b^
**TIBC (µg/dl)**	361.0 ± 52.9 (362.5)	364.7 ± 55.1 (365.0)	372.9 ± 48.9 (366.0)	0.163^a^
**Ferritin (ng/mL)**	56.6 ± 42.4 (45.0)	34.6 ± 21.3 (30.5)	32.7 ± 23.8 (25.5)	0.002^b^
**Vitamin B_12_ (pg/mL)**	436.2 ± 174.4 (422.7)	411.6 ± 196.7 (390.5)	361.6 ± 142.5 (366.5)	<0.001^b^
**Folate (ng/mL)**	10.9 ± 3.4 (10.7)	9.2 ± 4.3 (8.4)	9.7 ± 5.6 (8.5)	0.001^b^

SAA (Serum amyloid A), TIBC (Total iron binding capacity), SD (Standard deviation).

a Repeated Measure Anova Analysis.

b Friedman analysis.

Although vitamin B_12_ values decreased in the third month of the study, no levels that could be considered a deficiency were detected. At the end of the sixth month, vitamin B_12_ levels that could be considered deficient were measured in five patients. Following these results, the patients were started on vitamin B_12_ supplements by oral route (500 mg/day for eight weeks).

## Discussion

This prospective cohort study is the first pediatric research investigating the impact of colchicine use on micronutrient levels in newly diagnosed FMF patients. The surprising result of the study was the statistically significant decrease in the levels of vitamin B_12_ and folate, as well as the decrease in platelet count, ferritin and SAA, which were markers of inflammation, with the use of colchicine. Another surprising result was that 10.8 % of the patient group in whom the effect of colchicine was observed had vitamin B_12_ levels that met the definition of deficiency in the sixth month of treatment.

Colchicine is the gold-standard therapy for extending the duration between attacks, reducing the severity of the attacks, and indirectly improving anemia associated with chronic disease [4]. Korkmaz et al. assessed hemoglobin levels in patients using colchicine for at least six months, finding a significant increase in hemoglobin levels [10]. Meanwhile, Celkan et al. [11] reported that anemia was present in 63.4 % of newly diagnosed FMF patients. The frequency of anemia was higher compared to healthy controls, and they noted improvement in hemoglobin levels following colchicine treatment. Their patients' pre-treatment mean ferritin levels were elevated, indicating inflammation. As inflammation subsided with colchicine treatment, ferritin levels decreased to normal limits. In this study, the significant increase in hemoglobin levels and the decrease in ferritin levels after the introduction of colchicine treatment were consistent with the findings of Celkan et al.

Vitamin B_12_ and folate are micronutrients that mainly affect all age groups' hematopoietic and neurological systems. To the best of our knowledge, this is the first pediatric study to reveal a significant reduction in vitamin B_12_ and folate levels by comparing the pre-treatment period with the 3^rd^ and 6^th^ months of treatment. In a published animal study, colchicine has been shown to cause a reversible decrease in dose-dependent intrinsic factor-vitamin B_12_ receptor amount and leading to vitamin B_12_ deficiency [12]. In a distinct experimental study involving guinea pigs, colchicine was reported to induce malabsorption by reducing the number of intrinsic factor–vitamin B_12_ receptors in the intestinal mucosa [13]. Alten et al. [14] reported that mucosal injury in colchicine-treated FMF patients was attributed to a histopathological pattern characterized by crypt hyperplasia and villous atrophy, associated with increased cellular turnover. Webb et al. [15] also argued that oral vitamin B_12_ absorption in patients using colchicine decreased, especially in the ileum. However, there are also studies whose results have been determined in quite different directions. Ehrenfeld et al.’s [16] study of 20 patients with long-term colchicine use showed no reduction in vitamin B_12_ levels in the adult FMF population. Gemici et al. [17] compared 95 adult FMF patients with a mean duration of 10 years of colchicine therapy to 90 healthy controls. According to their findings, the prevalence of vitamin B_12_ deficiency was roughly fourfold higher in FMF patients compared to controls, yet the difference did not reach statistical significance. The authors nevertheless highlighted that vitamin B_12_ deficiency occurred predominantly in patients receiving long-term daily colchicine treatment. In a recent study comparing 333 children with FMF and 161 healthy children, vitamin B_12_ levels were reported to be markedly lower in the patient group, but levels that could be considered as deficient were not recorded [18]. In the present study, the authors found a statistically significant decrease in vitamin B_12_ levels between pre-treatment and the third month, and between pre-treatment and the sixth month. By the end of the sixth month, the authors recorded levels that met the definition of vitamin B_12_ deficiency (< 200 lt; 200 pg/mL) in five of the studied patients. In a study by Ekinci and colleagues that evaluated 63 children diagnosed with FMF, it was shown that 60 % of the patients had low serum vitamin B_12_ levels [19].

Folate, another key cofactor in erythropoiesis, may also be depleted in chronic inflammatory states. Luketic et al. [20] suggested that the decreased folate activity with colchicine and the anti-mitotic effect of colchicine were in a folate-dependent way. Similar to the literature, the present study indicated that serum levels of folate decreased by the third month of colchicine treatment and continued to decline by the sixth month of follow-up.

This study has several limitations that warrant acknowledgment. First, the assessment of patients’ dietary intake was limited to documenting the types of foods consumed (e.g., meat, nuts, and leafy green vegetables), without standardization of portion sizes; consequently, quantitative evaluation of intake was not feasible. This may influence serum levels of vitamins and iron, potentially confounding the assessment of micronutrient status. Second, due to the limited duration of follow-up, the long-term effects of colchicine therapy on micronutrient concentrations could not be fully assessed.

Nevertheless, the study has notable strengths. Most importantly, it employed a longitudinal design, allowing for intra-individual comparisons over time. Micronutrient levels were assessed both prior to the initiation of colchicine therapy and during treatment, providing valuable insights into the potential impact of colchicine on vitamin B_12_, folate, and iron homeostasis in children with FMF.

## Conclusion

This prospective study provides novel insights into the short-term impact of colchicine therapy on micronutrient status in children with newly diagnosed FMF. While colchicine effectively suppressed inflammation and led to significant improvement in hemoglobin levels, a concurrent and progressive decline in serum vitamin B_12_ and folate levels was observed over the 6-month follow-up. Notably, vitamin B_12_ levels dropped to a deficient range in five patients by the sixth month, underscoring the need for careful monitoring of micronutrient balance during treatment. These results emphasize that optimal FMF management should integrate nutritional evaluation into routine follow-up, ensuring comprehensive care that addresses both inflammatory control and metabolic health in pediatric patients.

## Funding

No funding was received for this study.

## Authors’ contributions

Conceptualization: ÖK, MÇ. Investigation: ÖK, AT. Writing – Original Draft: ÖK. Writing – Review & Editing: MÇ, ŞGK, NAA. Data Curation: ÖK, AT, RK. Formal Analysis: ÖK, MÇ, AT. Supervision: MÇ, NAA

## Data availability

The data that support the findings of this study are available from the corresponding author upon reasonable request.

## Conflicts of interest

The authors declare no conflicts of interest.
